# Presentation of Novel Architecture for Diagnosis and Identifying Breast Cancer Location Based on Ultrasound Images Using Machine Learning

**DOI:** 10.3390/diagnostics11101870

**Published:** 2021-10-11

**Authors:** Yaghoub Pourasad, Esmaeil Zarouri, Mohammad Salemizadeh Parizi, Amin Salih Mohammed

**Affiliations:** 1 Department of Electrical Engineering, Urmia University of Technology (UUT), Urmia 57166-93188, Iran; 2 School of Electrical Engineering, Electronic Engineering, Iran University of Science and Technology—IUST, Tehran 16846-13114, Iran; esmael_zaroori@elec.iust.ac.ir; 3 Department of Biomedical Engineering, University of Houston, Houston, TX 77204, USA; M.Salemizadeh_Parizi@hotmail.com; 4 Department of Computer Engineering, College of Engineering and Computer Science, Lebanese French University, Erbil 44001, Iraq; a.salihmohammed@outlook.com; 5 Department of Software and Informatics Engineering, Salahaddin University, Erbil 44002, Iraq

**Keywords:** breast cancer, deep learning, classification, segmentation, convolutional neural network

## Abstract

Breast cancer is one of the main causes of death among women worldwide. Early detection of this disease helps reduce the number of premature deaths. This research aims to design a method for identifying and diagnosing breast tumors based on ultrasound images. For this purpose, six techniques have been performed to detect and segment ultrasound images. Features of images are extracted using the fractal method. Moreover, k-nearest neighbor, support vector machine, decision tree, and Naïve Bayes classification techniques are used to classify images. Then, the convolutional neural network (CNN) architecture is designed to classify breast cancer based on ultrasound images directly. The presented model obtains the accuracy of the training set to 99.8%. Regarding the test results, this diagnosis validation is associated with 88.5% sensitivity. Based on the findings of this study, it can be concluded that the proposed high-potential CNN algorithm can be used to diagnose breast cancer from ultrasound images. The second presented CNN model can identify the original location of the tumor. The results show 92% of the images in the high-performance region with an AUC above 0.6. The proposed model can identify the tumor’s location and volume by morphological operations as a post-processing algorithm. These findings can also be used to monitor patients and prevent the growth of the infected area.

## 1. Introduction

Ultrasound is the main procedure for breast cancer detection and statistical analysis of results during a mechanical investigation. Ultrasound monitoring shifts the pathophysiology of breast cancer far from the most part massive lesions that are easily seen and effectively evident and toward ever smaller and occasionally harmless tumors [1]. A breakthrough in systems’ capacity to apply machine learning (ML) approaches to tackle a range of therapeutic scanning issues has occurred during the last decade. While straightforward computer-aided diagnosis (CAD) technologies have been in ultrasound for several years, their value and effectiveness have typically been restricted. New deep learning (DL) approaches, on the other hand, have been shown to identify cancers on standard mammograms as well as, if not superior to, professional physicians. It remains a challenge; the possibility of intelligence monitoring systems identifying autonomously in a randomized controlled trial has not been materialized. The current emphasis is on ML systems assisting radiologists instead of functioning as standalone diagnosticians [2]. Medical imaging, part of the broader scope of testing, is the biggest and most prospective channel via which DL may be utilized in healthcare [3,4]. To get a diagnosis promptly, radiographic examinations, despite modalities, need much interpretation by a professional clinician. There is a rising necessity for diagnostic automating as the constraints on existing radiologists increase [5,6]. Detecting malignancy in breast cancer images has previously been described using ML approaches. On the other hand, ML is restricted in interpreting essential information in its raw state. The constraint arises from the requirement for industry professionals who can manufacture information to feed a classification. On the other hand, DL, a branch of neural networks, learns several layers of description and conceptualization autonomously, allowing for a more in-depth analysis of breast cancer images. Artificial neural networks have made significant advances in image processing [7]. The prevalence of false positives is one of the issues connected with ultrasound. In Europe, women between the ages of 50 and 69 who undergo biannual screening face a 20% chance of receiving a false positive. The statistics in the U.S. are even more worrisome, with every tested woman experiencing at least one false-positive throughout her lifetime. The false-positive findings affect women’s lives, particularly in terms of daily welfare and medicine expenses. However, false positives are not ultrasound’s sole disadvantage [8]. Sure researchers have studied Nucleus analysis, who have extracted nucleus characteristics that can categorize cells as benign or malignant [9]. Likewise, grouping-based methods based on histogram equalization and various measurement characteristics have been used for nuclei recognition and classification. Nonetheless, the service’s effectiveness and efficiency suffer due to the complexity of traditional ML approaches like filtering, separation, and edge detection. The DL technique, which has just evolved, could solve standard ML problems. This technique can tackle picture identification and object localization problems with remarkable dimensionality reduction. CNNs are the most common DL algorithms available in the literature. The 2D input-image structure is used to modify the CNN architecture [10,11]. A CNN-training assignment needs a considerable amount of data in short supply in the healthcare field, particularly in BC. Using the TL method from a natural-images database, including ImageNet, and fine-tuning it answer problems.

In this paper, six analyses have been performed to detect and segment ultrasound images. Features of images are selected using the fractal method. After the k-nearest neighbor (KNN), support vector machine (SVM), decision tree (DT), and Naïve Bayes (NB) classification techniques are used to classify images. Then, convolutional neural network (CNN) architecture was designed to be directly classified based on ultrasound images. Finally, a CNN model is presented to identify the location of the breast cancer lesion.

## 2. Literature Review

Data mining methods were utilized by Ganggayah et al. to create models for discovering and displaying key prognostic markers of breast cancer survival ratio. There were 23 predictor factors in the database and one outcome variable, which alluded to the participants’ survival state. For performing firm using random forest, the information was grouped based on the receptor status of women with breast cancer detected by immunohistochemistry. The discovered key prediction variables impacting breast cancer survival rates, confirmed by survival curves, are helpful and may be converted into medical diagnosis systems [12]. To identify the Wisconsin Breast Cancer (Basic) database, Bayrak et al. employed two among the most prominent machine learning algorithms and evaluated their recognition accuracy. The Support Vector Machine method produced the most remarkable results with minor errors [13]. Zeebaree et al. presented a technique for extracting the region of interest (ROI) for detecting breast cancer abnormalities. The suggested model was developed using a local scanning technique and a classification technique. A learned simulation was performed in the learning phase by estimating the frequency of rounds from both ROI and environment. The background detected the ROI by monitoring the image with a set size window during the testing step. The suggested solution’s functionality was also matched to current techniques for segmenting specific inputs [14]. Using WDBC database, Agarap compared six ML methods by assessing their diagnostic quality standards. The hyper-parameters utilized for all the classes were control of work to construct the neural networks. According to the statistics, all the supervised learning models scored well on the created plan. With a test accuracy of 99.04 percent, the MLP strategy stands out between modeling and analysis [15]. Ferroni et al. demonstrated the value of combining an ML-based recommender system with stochastic optimization to retrieve diagnoses from frequently gathered breast cancer survivors’ personal, clinical, and molecular data. With a hazard ratio of 10.9, the algorithm could also screen the testing collection into people diagnosed with low- or high-risk advancement. Verification in revolutionary change trials was required, as was successful planning of security issues connected to computerized e-health data. Furthermore, the findings revealed that incorporating ML methods and models into e-health data might aid in obtaining therapeutic targets and could change the treatment of customized therapy [16]. Binder et al. demonstrated a machine-learning technique for comprehensively assessing phenotypic, biochemical, and clinical characteristics from breast cancer pathology that was easily understandable. Initially, the method enabled the accurate detection of tumors and tissue lymphocytes in pathological images and exact heat map representations that explained the classifier’s conclusions. Next, histology was used to identify molecular characteristics such as DNA methylation, gene expression, copy number changes, somatic mutations, and proteins. Eventually, using knowable AI, researchers determined the relationship between morphological and molecular cancer characteristics. Across a combined clinical score of histological, clinical, and molecular characteristics, the resultant statistical multiplex-histology model can help boost fundamental biomedical research and accuracy treatment [17]. There are metaheuristic algorithms such as Harris hawk’s optimization [18], multi-swarm whale [19], moth–flame optimizer [20,21,22], grey wolf [23,24], fruit fly [25,26], bacterial foraging optimization [27], Boosted binary Harris hawk’s optimizer [28], ant colony [29,30], biogeography-based whale optimization [31], and grasshopper optimization [32].

Souri et al. used ML to connect the activity of enzymes to overall survival and categorize tumors into more or less aggressive prediction types using breast cancer transcriptomics from numerous research projects. The proposed approach can categorize cancers into better-defined prognostic groupings instead of using knowledge on tumor volume, staging, or subtypes. The process helps increase prediction and enhance clinical decision-making and accuracy therapies, possibly reducing under diagnosis of high-risk cancers and reducing overtreatment of low-risk disease [33]. The efficiency of traditional ML and DL-based techniques was tested by Boumaraf et al. They also helped categorize breast cancer in histological images by providing a visual explanation. Using ML-based approaches, three feature extractors are used to obtain several features, which are then fused to create a feature representation that can train based on classical classification. They use the transfer learning technique to the VGG-19 classifier for DL-based approaches. They display the learned features after presenting the recognition accuracy of traditional ML and DL techniques to understand classification performance differences better. The results revealed that DL affected the cost ML methods [34]. To tackle the classification problem, Saxena et al. developed a new ML model.

The suggested model used pre-trained ResNet50 and the kernelized mixed deep neural network for CAD of breast cancer utilizing histology. The histological pictures of breast cancer were collected from massive databases. For the categorization of both minor and dominant class cases, the suggested approach performed relatively well. In perspective, the experimental result improves state-of-the-art ML models applied in prior research utilizing the identical BreakHis learning ratio [35]. Wang et al. presented a prototype transfer-generated adversarial network that combines generative adversarial systems and proto systems to categories a vast group of observations using a transfer learning classification model on a limited number of labeled input databases from a comparable area. This strategy decreased the pixel-level dispersion gap for breast histopathological images captured from different platforms with personality and style without necessitating a large number of labeled detection methods by generating an adversarial network, which decreased the style difference between the source and target. The pixel values learned by a prototype network were then embedded into the metric space, allowing discriminative information from the model to be extracted into the neural network. They trained an algorithm to predict huge quantities of target data using a specific “distance” in the subspace. The suggested approach for identifying benign and malignant tumors has an accuracy of almost 90%, according to the empirical results using the BreakHis sample. Shashaani et al. presented a new idea for detecting the effects of paclitaxel on normal and cancerous breast cells [36]. Nourbakhsh et al. demonstrated the effect of MDSC in autoimmune and its therapeutic application [37]. Khayamian et al. investigated the increase in cancer cell permeability and material absorption [38]. It demonstrates the benefit of our technique in offering a valuable tool for breast cancer multi-classification in healthcare situations while reducing the expense of complex annotation [39]. In addition, biological uses of computer vision are prevalent, for instance, diagnosis of tuberculous [40], thyroid Nodules [41], Parkinson’s [42], and paraquat-poisoned patients [43] (See Table 1).

## 3. Materials and Methods

### 3.1. Feature Extraction

Feature extraction aims to reduce the number of resources needed to depict an extensive set of data correctly. One of the most significant issues when doing complicated data collection is the number of factors studied. A high range of factors necessitates much memory and storage capacity, or a classifier that employs the instructive example and adapts to new situations. Feature extraction is a broad phrase that refers to strategies for putting together a set of variables to tackle high-precision issues. Image analysis aims to develop a unique approach to portray the essential elements of images in a particular way. A gray area vector was constructed in the fractal technique to produce feature vectors. The image characteristics of the confidence interval of the detected chemicals are computed in statistical analysis from the light intensity of the specified places relative to someone in the image. The frequency of intensity points (pixels) in each combination affects the statistics [53]. The fractal model is utilized to extract the feature in this work. Feature selection has been used to minimize the dimensions and find other fundamental characteristics that may sufficiently distinguish the different systems in engaging with high input data [54].

The fractal technique was used with covariance analysis to create eigenvalues from the image and lower the dimension. The input images for the fractal method must be the same size, and one image is referred to as a two-dimensional matrix and a single vector. Grayscale images with a specified resolution are required. By reshaping matrices, each image is transformed into a column vector. The photos are taken from a M×N matrix. *N* represents the number of pixels in each image, and *M* is the number of images. To determine the normal distribution of each original image, the average image must be computed. The covariance matrix would then be calculated, and the covariance matrix’s eigenvalues and eigenvector are produced. The fractal system’s method is that *M* represents the number of training images, $F_{i}$ is the mean of the images, and $l_{i}$ represents each image in $T_{i}$. There are *M* images at first, each of which has the N×N size. Each image may be presented in an N-dimensional area using Equations (1) and (2) for average operations [55].

(1) A=N×N×M

(2) $$F_{i}=\frac{1}{M}\sum_{t=1}^{m}Tt$$

The fractal method assigns the standard deviation as a critical issue, computed using Equation (3) and the covariance matrix in Equation (4).

(3) $$Variance=\frac{1}{M}\sum_{t=1}^{m}Tt$$

(4) $$Cov=AA^{T}$$

Such that $A=\lfloor Variance_{1},\;Variance_{2},\ldots ,\;Variance_{n}\rfloor$ and $Cov=N^{2}*N^{2}$ and $A=N^{2}\;*\;M$. Cov equals by a considerable value. Then the eigenvalues of Cov are found based on Equation (5).

(5) $$U_{i}=AV_{i}$$

Total scatter or covariance matrices are computed using Equations (6) and (7) for scattered matrices in the subclass [55].

(6) $$S_{T}=\overset{N}{\underset{k=1}{\sum }}\left(x_{k}-\mu \right){\left(x_{k}-\mu \right)}^{T}$$

(7) $$W_{Fractal}=arg\;max\left[W^{T}S_{T}W\right]=\left[w_{1}w_{2}\ldots \;w_{f}\right]$$

μ is the average of all data and $\left\{w_{i}\;|\;i=1,2,\ldots ,\;f\right\}$ is a set of eigen vector of f-dimension of $S_{T}$ that is associated with the largest eigenvalue f.

### 3.2. Convolutional Neural Network (CNN)

The CNN technique is explained in this section. One of the learning networks motivated by the Perceptron neural network is this sort of neural network. An input layer, an output layer, and a hidden deep layer make up this deep network. Initially, the issue’s images or data are classified and taught into the method. The weights of the hidden output layer might then appear in a variety of ways. The suggested method is a classifying or recognition approach if the algorithm’s output comprises many quantitative elements such as a binary or score. The given process is segmentation or identification if the output layer is a matrix as the input image as ground truth information. Convolutional neural networks (CNNs) are composed of convolutions, resampling, and fully coupled layers. The three head neuronal layers are convolutional, pooling layers, and fully associated layers [56]. Each layer has a different task assigned to it. Feature extractor layers are made up of convolutions and subsampling layers [57,58].

In contrast, a related layer order that classifies current data has a place using separated features. The components of feature maps and predictive utility are limited when a pooling layer is assigned. Because the computations of pooling layers consider nearby pixels, these change invariantly. The system is prepared using both forward and regressive progress. The forward progress aims to define the information image using the current parameters (loads and inclination) [59,60].

### 3.3. Performance Analysis Criteria

On a different collection of samples, called a test set, we examined the performance of a classifier. The accuracy rate is the standard evaluation metric in DL; accuracy correctly classifying the percent of test samples. The loss function is measured as the ratio of incorrectly categorized test samples divided by the total number of test samples. Therefore, records with a significant number of occurrences of one class compared to another are inappropriate for accuracy in an imbalanced dataset. Unless the issue has an imperfect model, a classifier that consistently identifies the majority class regardless of information is highly accurate. We utilize classification confusion matrix-based criteria in extracted features. The outcomes of a predictor in the training dataset are summarized in a confusion matrix. False positives anticipate many negative tests that are surprisingly positive. In contrast, true positives regard a quantity of positively predicted positive samples to be positive. True and false negatives are both based on the same principles. We could construct some important ones using the confusion matrix [60]:

(8) $$sensitivity=\frac{TP}{TP+FN}$$

(9) $$Percision=\frac{TP}{TP+FP}$$

(10) $$Accuracy=\frac{TN+TP}{FP+TN+FN+TP}$$

The anticipated positive sample ratio’s sensitivity is positive, indicating that the expected negative sample ratio is also negative. The projected data set accuracy is positive and was positive. High sensitivity and specificity, or high accuracy and specificity, are both characteristics of a successful categorization. Sensitivity and specificity are desirable in diagnosing diseases, but accuracy and sensitivity are favored in ML. The chance that a sample is categorized as positive is the classification criteria. It strikes a balance between sensitivity and property (or, equivalently, accuracy, and evocation): a low-threshold training set is susceptible to classifying samples as positive, but it also has the potential to produce a large number of false positives, so it has high sensitivity but a low feature, etc. for high thresholds. The recall curve for an exact piece’s categorization differs from its recall as a threshold.

## 4. Results and Discussion

### 4.1. Data Collection

The data collected initially included breast ultrasound images of women between the ages of 25 and 75. This data was organized in 2018. The number of patients is 400 women. The data set contains 780 images with an average image size of 500 by 500 pixels. Images are in PNG format [36]. Images are classified into three classes: normal, benign, and malignant. In this research, for image classification and segmentation, the image size has been reduced to 256 by 256 to reduce the processing complexity.

### 4.2. Ground Truth Images

**Figure 1:**
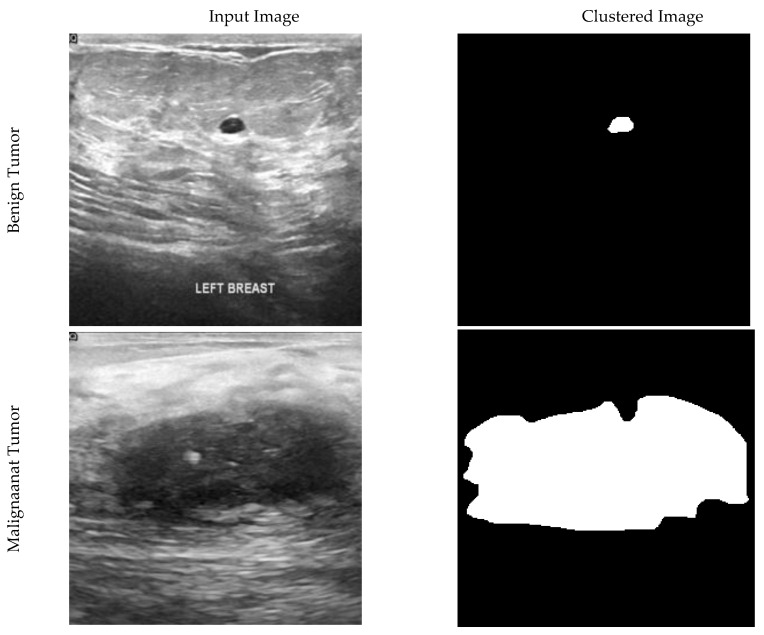

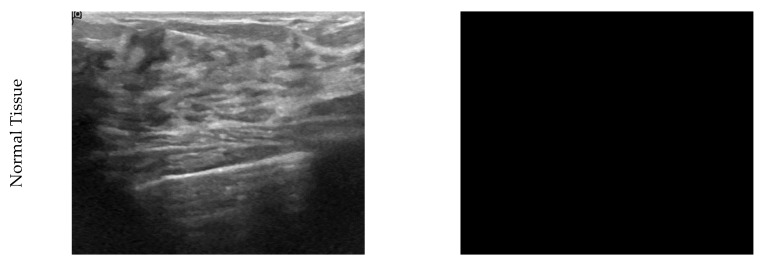
Ultrasound and clustered images of breast cancer for benign, malignant, and normal tissue conditions.

In this paper, segmentation of images has been used to find the primary location of the tumor. Segmentation is not one of the main steps of the convolution and DL algorithm. It has been used to validate the results. By separating pixels with zero values as the background, each non-zero pixel is the mass breast threshold (225). Each remaining pixel is 127 to the normal breast tissue, as shown in Figure 1.

Ultrasound has higher images quality and does not have any marks or scan effects on film; this allows the network to learn more specific features and segmentation. Having many images increases the model’s accuracy by increasing your data set and training on overlapping pieces. Eighty percent of the images are randomly assigned to the training sets and 20% to the test set for each division.

### 4.3. Feature Selection

**Figure 2:**
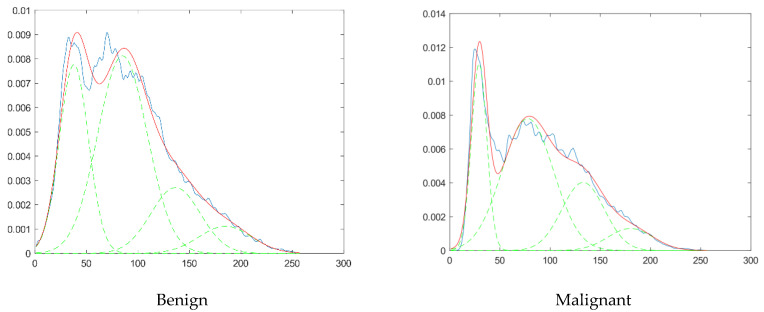
Histogram of features extracted from the ultrasound images. Blue: Histogram of the image, Red: Modeled histogram, Green: Gaussian Functions.

Fractal features extracted from ultrasound images are used in model classification. In the fractal method, the histogram of the images on the images is extracted, as shown in Figure 2.

According to the diagram in Figure 2, the images are transformed to a histogram and modeled by the fractal method. As a result, the obtained model is replaced by four graphs of the normal distribution function. The characteristics of the obtained distributions are obtained in the form of four numbers as features of each image. The features are stored in a matrix and ready to be classified. Figure 2 shows the blue line of the image histogram. By rearranging the images used for modeling, we selected four features with higher accuracy. A red line indicates the sum of the functions. Image features are parameters of Gaussian functions. This process is done for all data set images. Thus, the classification data set is converted to a matrix using four features.

### 4.4. Classification of Ultrasound Images by Traditional Methods

In this part, the results of CNN architecture in breast cancer ultrasound images are presented. The fractal method is one of the most potent methods and feature selection in images, especially MRI; cancer has been diagnosed by combining these features and famous classifications. Feature extraction output for each image is four scalars, which are used as classification inputs. Moreover, the outer layer of all classifications was labeled 0 for normal tissue, 1 for benign tumors, and 2 for malignant tumors. The proposed models are designed to diagnose cancer types. The classification outputs are plotted as confusion matrices.

**Figure 3:**
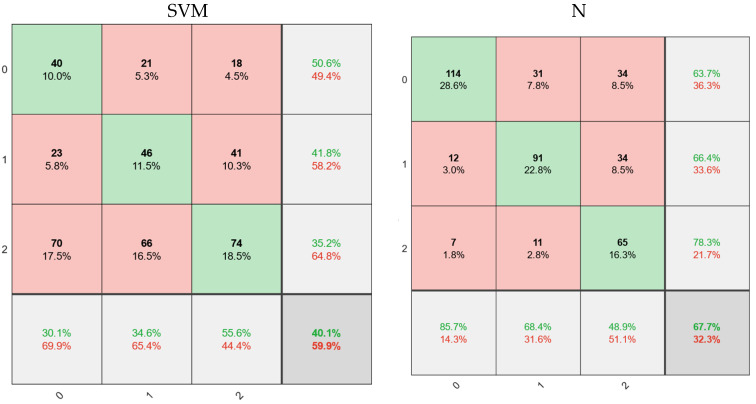

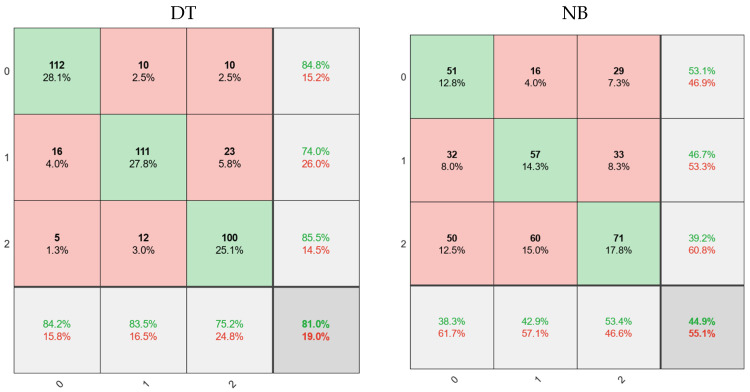
Confusion matrices for classifying or diagnosing tumor type and disease.

According to Figure 3, green cells show true values, and red cells show the number of images with false results. Gray cells also show sensitivity values (horizontal) and precision (vertical). Finally, the more colorful cell in the left corner estimated the total accuracy for the different models. According to the figure, four classical classification models have been selected to detect the type of tumor, among the most powerful methods for diagnosis and classification. These classifications usually give perfect results for binary detection. However, they have many problems for multiple classifications (for example, triplets in this case). According to the results of the decision tree method with an acceptable amount can help diagnose cancer. Out of 133 images with normal tissue in this method, 122 images (84.2%) were correctly diagnosed. In addition, 111 images (83.5% of patients) with benign tumors were correctly diagnosed. In this category of education, 133 images were correctly identified. With benign tumors, 10 images were diagnosed as healthy and 12 as malignant, of which 22 false images were recorded. Finally, the accuracy of the decision tree method was 81%, and the error rate was 19%. Following KNN, SVM and NB were recorded with 67.7%, 40.1%, and 44.9% accuracy, respectively. This level of accuracy could not satisfy the classification with complete accuracy. Therefore, we need to design a model that can diagnose the disease more accurately and sensitively. Therefore, in the next section, the proposed model based on a convolutional neural network is presented.

### 4.5. Classification of Ultrasound Images Based on Presented CNN Method

In this section, the results of CNN architecture in breast cancer ultrasound images are presented. In this classification, there are three classes: category of benign images, malignant images, and healthy or normal tissues. The CNN architectural model is trained using dataset images, and evaluation criteria are performed to analyze the model, which is as follows.

**Figure 4:**
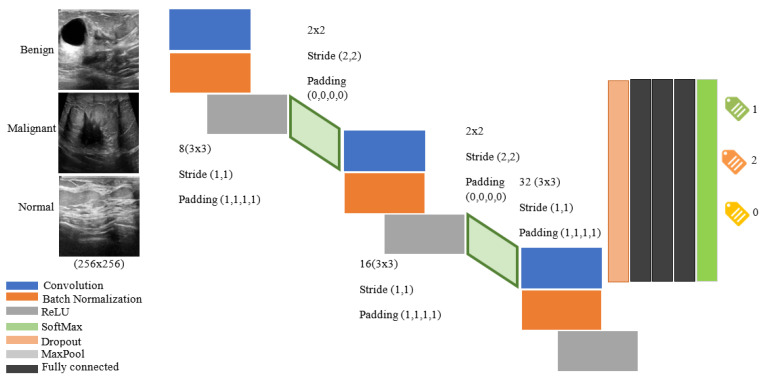
The architecture provided by CNN for classifying or diagnosing tumor type and disease.

**Figure 5:**
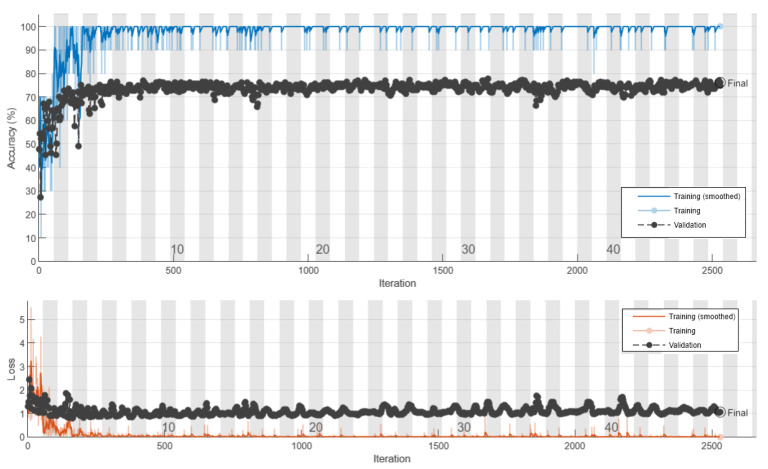
The accuracy and loss of the CNN classification model during program execution.

Figure 4 shows the architecture of CNN’s proposed methods for diagnosing cancerous tumors. This network consists of 16 layers with three layers of convolution. Images have labels for patients with a benign tumor equal to 1 malignant equal to 2 and healthy individuals zero. In addition, 70% of the image datasets are used for network training and 30% for model testing. The results are shown in the following section. Figure 5 shows the amount of loss and accuracy as a function of training epochs. These diagrams are depicted during network training. The process ended after obtaining the best result with higher accuracy and less network loss for 3000 iterations. The back points show the cross-validation of the training process.

**Figure 6:**
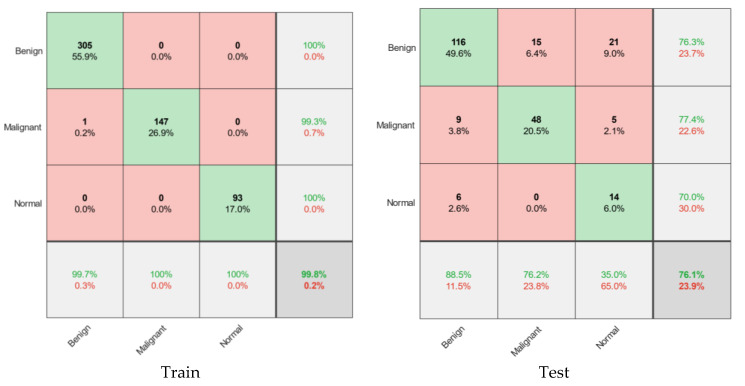
The confusion matrix for the training and test set for the proposed CNN model.

The final trained model is evaluated in both training and experimental sets. Figure 6 of the confusion matrices shows this prediction. As can be seen in this figure, the model with 306 images of benign tumors properly trained 305 items (99.7%) in the training set. In addition, out of 147 malignant images and 93 healthy tissue images, 100 images are accurately predictable. As a result, the accuracy of the training set is 99.8%. The results are as follows in the experimental group, which is 30% of the original data and did not participate in the modeling.

**Figure 7:**
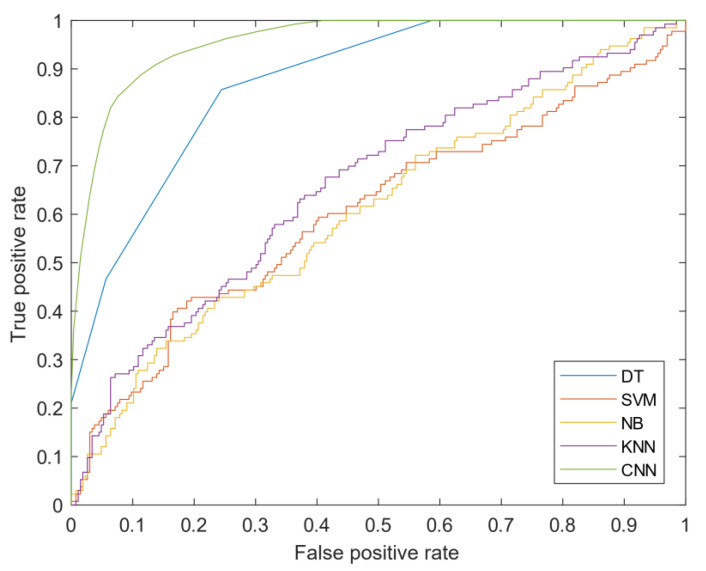
ROC curve for the various classifications presented in the research.

Regarding the test results, out of 131 benign experimental images, 116 images were correctly detected. In other words, this diagnosis was associated with 88.5% sensitivity. In addition, out of 63 malignant images, 48 images (76.2%) were correctly diagnosed, of which 15 images (23.8%) were misdiagnosed as benign, which are called false results. This model was associated with low sensitivity or 35% to identify healthy tissues that did not participate in the model. In other words, the total accuracy of the model for the validation of the proposed model is 76.1%. The results of comparing the proposed models for cancer diagnosis are presented in Table 2 and Figure 7. According to Table 2, the model presented by CNN diagnosed cancer with much higher accuracy, which has performed better than other methods and provided significant improvement. According to the receiver operating characteristic (ROC) diagram of Figure 7, the area under the ROC curve (AUC) is another measure of the efficiency of the classification models, which achieved 96% for the proposed model. Based on the findings of this study, it can be concluded that the proposed high-potential CNN algorithm can be used to diagnose breast cancer from ultrasound images. In the next section, we present a segmentation method for detecting tumor tissue.

### 4.6. Segmentation of Ultrasound Images Using the Presented CNN

**Figure 8:**
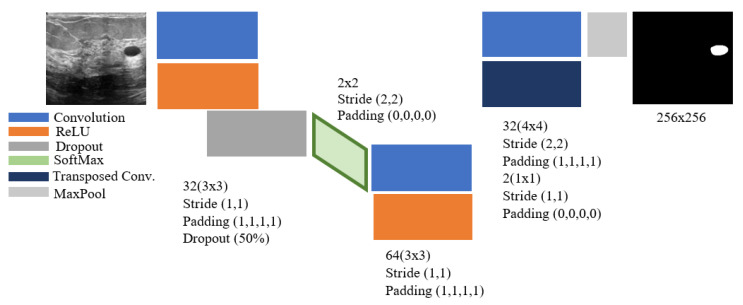
Presented CNN network architecture for the cancer tumor segmentation.

In this section, we present the results of the CNN segmentation algorithm. The architecture of the detection method is shown in Figure 8. It consists of 11 layers with three layers of convolution. Input images 256 × 256 are breast cancers, and the output layer contains ground truth or labeled input images. The number 255 is labeled on the tumor tissue in these images, and the other points are shown with the number zero. The proposed network is a kind of classification network. The output image pixels are selected as classification labels instead of the image label itself. In other words, the segmentation monitored in this study is a kind of classification with higher dimensions to classify the pixels and detect the infected area. Naturally, supervised segmentation is one of the most complex image processing issues in deep learning, which requires higher processing time.

**Figure 9:**
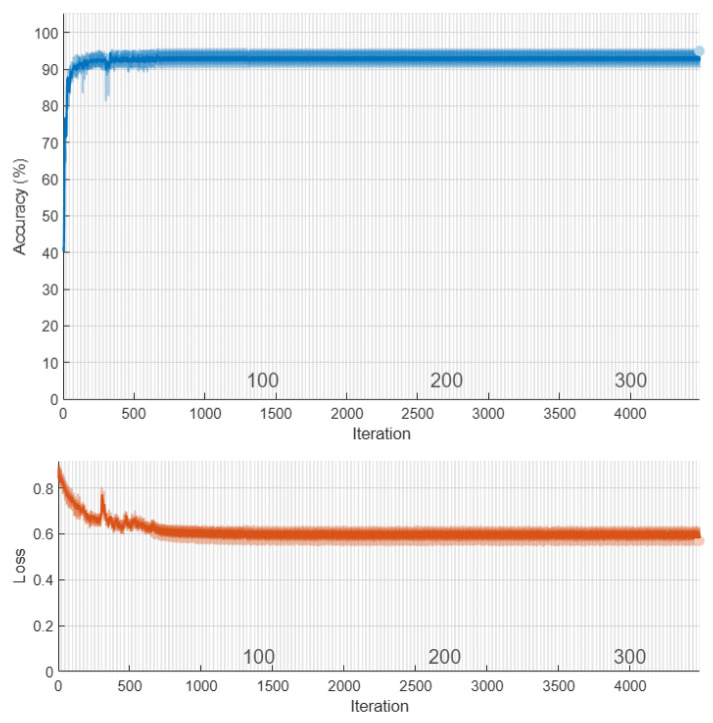
The accuracy and loss of CNN network for cancer tumor segmentation.

In the input image in Figure 8, the gray area shows healthy human findings, with part of the image appearing in a darker state. In classifying or segmenting images, the input image is meaningful for the model. In other words, the presence of high dark pixels in the model indicates that these lesions are also tumors, while the tumor part is seen as round. Accordingly, the images should be such that they can evaluate the tumor diagnosis with higher accuracy. Recognizing this area using computer image processing is challenging. Due to the potential of deep learning methods for dividing areas with different colors is more than images with almost the same color. In this study, the infected area is first identified and labeled by a physician or automated algorithm. Therefore, ground truth images consisting of the tumor area are located in the architectural output layer. The algorithm has been trained with 5000 iterations, of which 70% of the data is used for model training and 30% for testing the proposed model. The amount of accuracy and loss of CNN model training provided in Figure 9 is shown.

**Figure 10:**
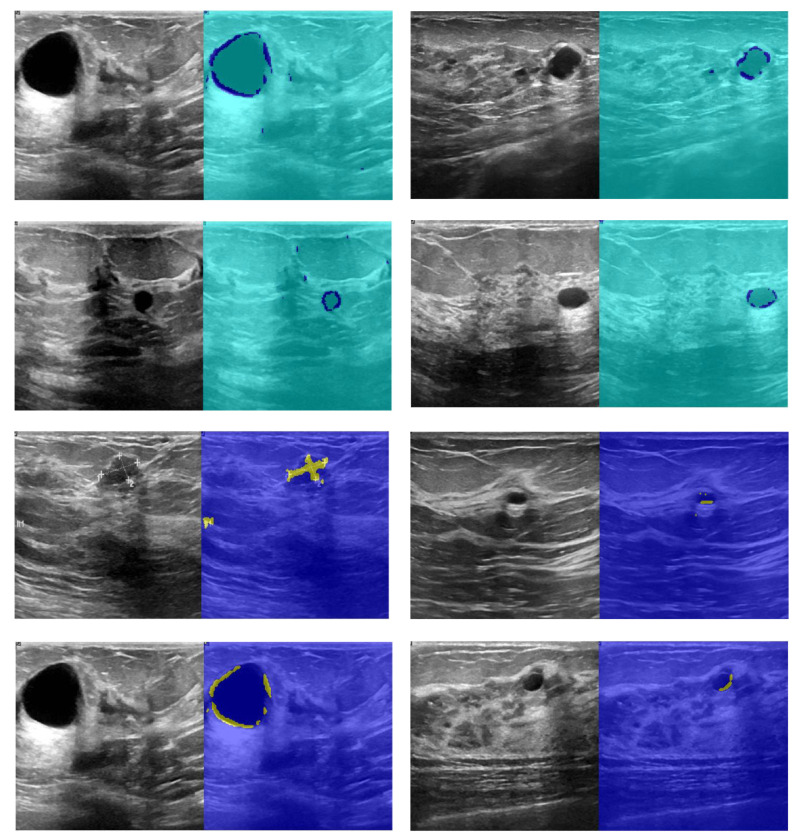
Results of CNN network cancer tumor segmentation.

**Figure 11:**
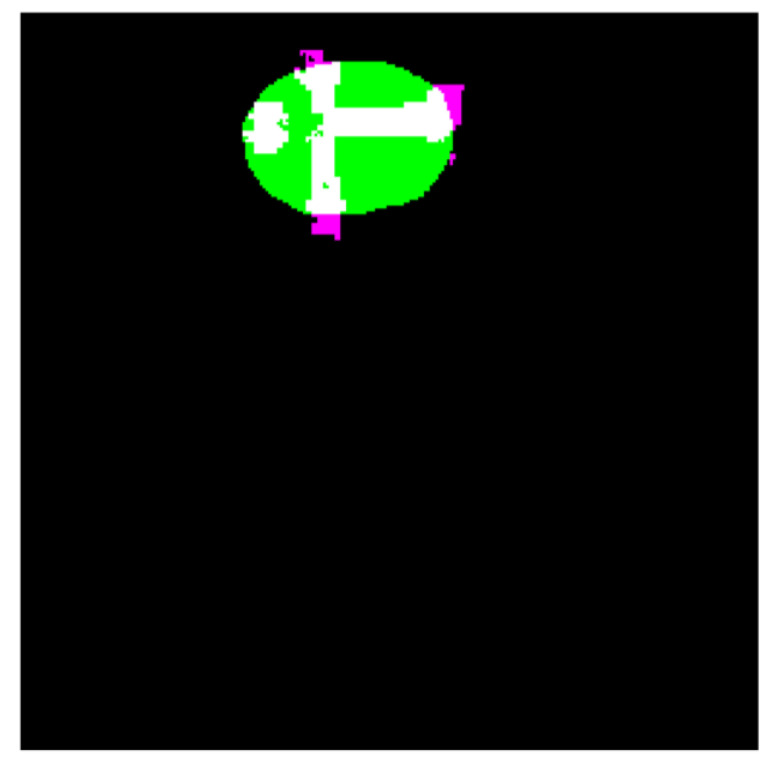
The results of CNN network cancer tumor morphology operations.

The segmentation results are shown in Figure 10. According to Figure 10, the first and third columns of the input image show the image infected with the cancerous tumor. Moreover, the other side of the image shows the segmentation results. Seventy percent of the images used for network training and 30% for test results start the process. The results of detecting the infected area are shown in the second and fourth columns of Figure 10. The resulting images should look like ground truth images. According to the results, the presented findings are almost similar to the model output. They have correctly identified the location of the tumor. To better increase the output, minor points in the results should be connected with morphological operations. Because the output points of the model were able to identify the approximate location and size of the tumor, we relate the tumor morphology to the original size of cancer. Figure 11 shows the approximate location and size of the tumor after morphological surgery. The results show that the proposed architecture can correctly identify the contaminated area.

**Figure 12:**
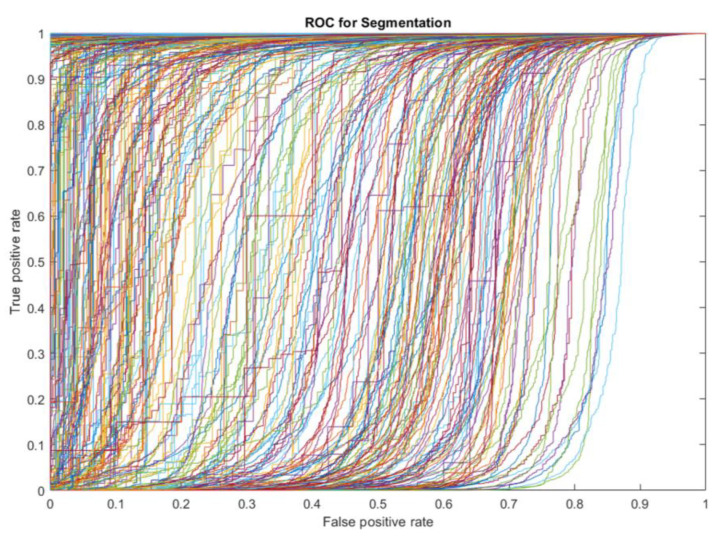
The ROC curve of a CNN network cancer tumor.

**Figure 13:**
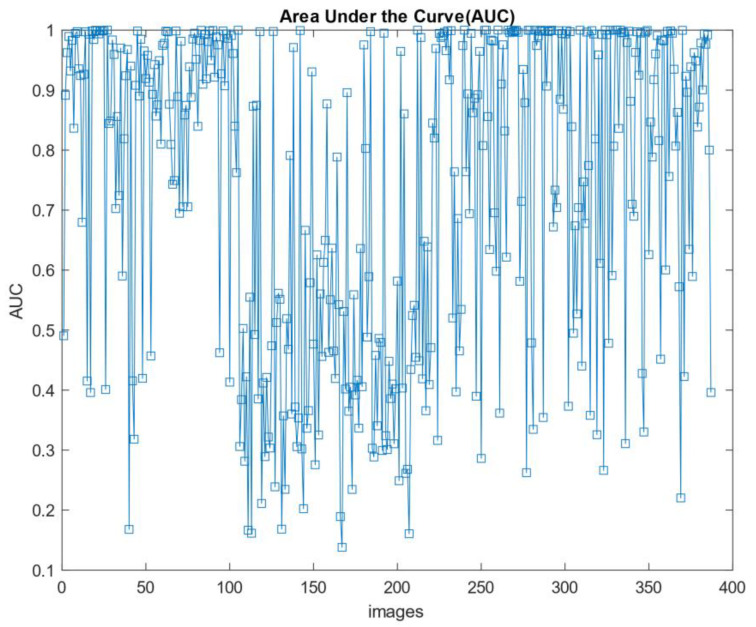
AUC value plot for images used on CNN.

The results of the segmentation method are presented, theoretically, with performance criteria. The segmentation criteria are almost different from the classification methods shown in the previous section. The fusion matrices are used for classification. If drawn, a matrix in the fusion must be drawn for all images. Accordingly, the ROC curve with a true positive rate versus a false positive rate is the best criterion for evaluating the model. This criterion is unique to each image in the segmentation algorithm. The high-performance image trend is correct whether the graphs are shown with a higher true positive rate and a lower false-positive rate. The results show that the maximum number of ROC curves below the higher efficiency graph of the model’s high efficiency shows. According to Figure 12, to understand the ROC curve with specific values, we present the area under the curve (AUC). This measure shows the high performance of the model for each image. In this section, 400 images of benign breast cancer are included in the model. According to Figure 13, 92% of the images in the high-performance region with an AUC above 0.6. According to the graph, the high AUC shows the segmentation of the images in true mode, and the low AUC offers the detection of pixels in false mode. The proposed model results identified the tumor’s location and volume by morphological operations as a post-processing algorithm.

## 5. Conclusions

Breast cancer is one of the leading causes of death among women worldwide. Early detection helps reduce the number of premature deaths. This study uses medical ultrasound scans to examine medical images of breast cancer. Breast ultrasound datasets are classified into three classes: normal, benign, and malignant. When combined with ML, breast ultrasound images can have great results in classifying, diagnosing, and classifying breast cancer. This study presents six ML methods for classifying and segmenting ultrasound images of CT scans of cancer patients. Six ML methods have been performed to detect and segment ultrasound images to diagnose the disease or tumor type. First, the features of the images are extracted using the fractal method. Then KNN, SVM, DT, and NB classification techniques were used to classify patients’ images. Then, the convolution neural network (CNN) architecture was designed to classify patients based on direct ultrasound images. Traditional classifiers provide excellent results for binary recognition but have many problems for multiple classifications. According to the decision tree method or DT, results with an acceptable amount can help diagnose cancer.

The accuracy of the decision tree method is 81%, and the error rate is 19%. Following KNN, SVM and NB were recorded with 67.7%, 40.1%, and 44.9% accuracy, respectively. The final model trained in both the training and experimental sets for the proposed CNN method is evaluated. The presented model trained 305 cases (99.7%) correctly in 306 images with benign tumors. As a result, the accuracy of the training set is 99.8%. Regarding the test results, out of 131 benign experimental images, 116 were correctly detected; in other words, this diagnosis was associated with 88.5% sensitivity. In other words, the total accuracy of the model for the validation of the proposed model is 76.1%. Based on the findings of this study, it can be concluded that the proposed high-potential CNN algorithm can be used to diagnose breast cancer from ultrasound images. The second CNN model presented was able to identify the original location of the tumor. The results show 92% of the images in the high-performance region with an AUC above 0.6. The proposed model results identified the tumor’s location and volume by morphological operations as a post-processing algorithm. These findings can also be used to monitor patients and prevent the growth of the infected area. Much work is being done to classify patients using artificial intelligence, such as diagnosing brain tumors, breast cancer, and lung cancer. However, implementing these approaches is not always convenient. These methods can be used in a wearable monitoring system to diagnose the disease, monitor, and transfer to specific physicians. According to studies on ML in medical image processing, it is time to implement artificial intelligence methods in medicine to help physicians make better diagnoses and as soon as possible.

## Figures and Tables

**Table 1:** Summary of research for diagnosis of breast cancer based on DL approaches.

Author	Year	Type	Network	Results
Yu et al. [44]	2021	Auxiliary diagnosis	Inception-v3	Breast cancer diagnosis accuracy in distant locations has improved.
Jiang et al. [45]	2021	Assessment of molecular subtypes	DCNN	The DL algorithm uses pretreatment ultrasound images of breast cancer to identify molecular subtypes with excellent diagnosis accuracy.
Bychkov et al. [46]	2021	Identifying morphological feature	DNN	The success of adjuvant anti-ERBB2 therapy was linked to ERBB2-associated morphology, which might help predict treatment outcomes in breast cancer.
Saber et al. [47]	2021	Automatic Detection and Classification	ResNet50, VGG-16, Inception-V2 ResNet	Overall accuracy is 98.96%
Boumaraf et al. [34]	2021	Image Classification of Histopathological Breast Cancer concerning Magnification	VGG-19	The pathologist believes autonomous DL techniques as a legitimate and credible support tool for breast cancer detection can be enhanced by the decisions.
Lee et al. [48]	2021	Prediction of axillary lymph node metastases	CNN	The findings show that the suggested CAP paradigm, which includes primary tumor and peritumoral cells to determine ALN status in women with symptomatic breast cancer, is reliable for predicting the ALN condition.
Zhang et al. [49]	2021	Molecular Subtype Diagnosis	Optimized DL model	Furthermore, this model’s prediction capacity for molecular subtypes was good, which has therapeutic implications.
Zhou et al. [50]	2020	Lymph Node Metastasis Prediction	Inception V3, Inception-ResNet V2, and ResNet-101	Using ultrasound images from patients with initial breast cancer, DL algorithms can accurately predict clinically negative axillary lymph node metastases.
Sharma and Mehra [51]	2020	Histopathology classification	VGG16, VGG19, and ResNet50	For all magnification variables, the benign and malignant classes are the most complicated.
Hu et al. [52]	2020	Multiparametric MRI is used to diagnose breast cancer.	CNN	The multilayer perceptron transfer learning technique for MRI may boost prediction value in breast imaging interpretation by lowering the false positive rate and increasing the high accuracy rate.

**Table 2:** Comparison of different classification models.

Model	AUC	Error	Accuracy
Presented CNN	0.96	0.20%	99.80%
DT	0.87	19%	81%
KNN	0.66	32.30%	67.70%
SVM	0.6	59.90%	40.10%
NB	0.6	55.10%	44.90%

## Data Availability

The Dataset of breast ultrasound images is available online https://www.kaggle.com/aryashah2k/breast-ultrasound-images-dataset (accessed on 3 October 2021).
